# Intrinsically Disordered Proteins Display No Preference for Chaperone Binding In Vivo

**DOI:** 10.1371/journal.pcbi.1000017

**Published:** 2008-03-07

**Authors:** Hedi Hegyi, Peter Tompa

**Affiliations:** Institute of Enzymology, Biological Research Center, Hungarian Academy of Sciences, Budapest, Hungary; McGill University, Canada

## Abstract

Intrinsically disordered/unstructured proteins (IDPs) are extremely sensitive to proteolysis in vitro, but show no enhanced degradation rates in vivo. Their existence and functioning may be explained if IDPs are preferentially associated with chaperones in the cell, which may offer protection against degradation by proteases. To test this inference, we took pairwise interaction data from high-throughput interaction studies and analyzed to see if predicted disorder correlates with the tendency of chaperone binding by proteins. Our major finding is that disorder predicted by the IUPred algorithm actually shows negative correlation with chaperone binding in *E. coli*, *S. cerevisiae*, and metazoa species. Since predicted disorder positively correlates with the tendency of partner binding in the interactome, the difference between the disorder of chaperone-binding and non-binding proteins is even more pronounced if normalized to their overall tendency to be involved in pairwise protein–protein interactions. We argue that chaperone binding is primarily required for folding of globular proteins, as reflected in an increased preference for chaperones of proteins in which at least one Pfam domain exists. In terms of the functional consequences of chaperone binding of mostly disordered proteins, we suggest that its primary reason is not the assistance of folding, but promotion of assembly with partners. In support of this conclusion, we show that IDPs that bind chaperones also tend to bind other proteins.

## Author Summary

Intrinsically disordered/unstructured proteins (IDPs) defy the classical structure–function paradigm because they exist and function without a well-defined 3-D structure. These proteins are extremely sensitive to degradation in the test tube, but show no enhanced degradation rates in the cell. To resolve this apparent contradiction, we tested whether IDPs are protected by interaction with accessory proteins, chaperones, often implicated in guarding other proteins in the cell. Our major finding is that disorder predicted by the IUPred algorithm actually shows negative correlation with chaperone binding in various species. To explain this finding, we argue that IDPs are protected in the cell from proteases by their special amino acid composition, and also by the tight regulation of intracellular proteases. Thus, the primary reason for their chaperone binding is not protection from degradation, but promotion of assembly with partners.

## Introduction

Intrinsically disordered/unstructured proteins or protein domains (IDPs) are prevalent in proteomes [1]–[3] due to the inherent functional advantages structural disorder imparts on proteins [1], [4]–[6]. *In vitro*, IDPs have been noted for an increased speed of interaction, specificity without excessive binding strength, adaptability to multiple partners and ease of regulation by post-translational modification. These and other functional features explain a particularly high level of disorder in important regulatory proteins involved in signaling, and regulation of transcription, such as p53 [7], p27^Kip1^ [8], CREB [9] or BRCA1 [10]. Whereas these features elucidate the prevalence of protein disorder in proteomes underlying the recent heightened interest in the subject, the phenomenon of structural disorder poses further serious questions. Due to their open and flexible conformational state, IDPs are exceptionally sensitive to proteolysis *in vitro* [4],[11], which raises concerns in terms of their *in vivo* existence and functioning. The question most often asked is how IDPs function when they are supposedly rapidly degraded by proteases *in vivo*. That this is not the case, is shown by our recent observations that the physiological half-lives of IDPs determined in a high-throughput study [12] show very weak correlation with their disorder content [13]. This suggests the involvement of additional factors and/or special mechanisms in the physiological function of IDPs, specifically addressed in this work. One particularly intriguing point is the possibility of the involvement of chaperones, which may offer direct protection by binding in the cell. Since chaperone action has already been implicated with some IDPs [14]–[16], we have decided to analyze recent high-throughput interaction data to provide a systematic and coherent answer to this question.

Chaperones are energy-dependent protein machines that function to prevent their clients from misfolding and aggregation, or to assist their assembly and transport in the crowded intracellular milieu [17]. Recently, it has been recognized that some IDPs also display chaperone activity, probably enabled by a more primitive mechanism that relies on “entropy transfer” from the chaperone to the misfolded partner [18]. Although in the original formulations chaperone models have been described as assisting folding of misfolded globular proteins and RNA molecules, in some cases it has been described that a chaperone may also have an IDP client. For example, it has been shown that molecular chaperones α(s)- and β-casein prevent amyloid fibril formation by κ-casein [15]. In another study, it was shown that chaperones promote the association of a microtubule-associated protein, tau, with microtubules [14]. The suppression of α-synuclein toxicity and aggregation in a Drosophila model for Parkinson's disease may also point towards the involvement of a chaperone in the action of an IDP [19],[20]. α-synuclein aggregation is also affected by another chaperone, αB-crystallin [16]. These examples show that some IDPs may require the involvement of chaperones for function, which could also explain the observed *in vivo* stability of these proteins. Whether this interdependence is general among IDPs, has been the subject of this study.

In recent high-throughput interaction studies large segments of the interactome, i.e. network of protein-protein interactions, have been described [21]–[23]. We have approached the above question by analyzing whether structural disorder correlates with the tendency of proteins to be binding partners of chaperones. We found that on the contrary, partners of chaperones tend to be ordered proteins, which apparently need more assistance for folding than IDPs. IDPs, on the other hand, need no help for folding, also suggested by many *in vitro* data on their functional efficacy, and probably use chaperone assistance for protection from aggregation and assembly into complexes.

### Data

We used the data about pairwise interactions published in the IntAct database (http://www.ebi.ac.uk/intact/site/index.jsf) [24]. It is a collection of interactions between proteins detected with various types of methods, culled from numerous publications and also databases such as the MSD, the Macromolecular Structure Database [25]. It contains system-wide interaction data regarding *E. coli* and yeast but only fragmented/partial interaction information about higher organisms. Complexes in the databases vary in size from two to more than a hundred components. Due to technical limitations, there is no information on the interaction of any two proteins within a complex of three components and above. Thus, to make sure our analysis focuses on the direct interaction of a protein with its partners, we selected complexes of exactly two components, regardless of the detection method, ensuring an actual physical interaction between the partners.

We grouped the interaction data in IntAct into three phylogenetic subgroups, handling the bacterial (mostly *E. coli*), unicellular eukaryotic (mostly yeast) and metazoa protein interaction data separately. The data reflects the status of IntAct as of December 6, 2006, which contained 729 bacterial, 7615 unicellular eukaryotic and 35,435 metazoa pairwise protein interactions (of the latter more than 24,000 were between *D. melanogaster*, 4,000 between human and more than 4,000 between *C. elegans* proteins).

## Methods

### Selecting chaperone-binding and non-chaperone-binding proteins

We identified chaperones among the interacting proteins based on their annotation in Swissprot and TrEMBL. However, we also identified “putative chaperones” by comparing all the interacting proteins with all the known chaperones in SwissProt and TrEMBL using Blastp [26] and designating a protein a putative chaperone if it had an at least 50% sequence identity and an almost full-length match (with the possible exception of 30 amino acids at either end) to any known chaperone. However, all the other proteins with a 50% or higher similarity (but not fulfilling the ‘almost full-length’ similarity) were excluded from both the chaperone and the non-chaperone class because of their perceived ambiguity regarding a chaperone function. To avoid false chaperone assignments among the short putative chaperones, we removed all the predicted chaperones with a length of less than 100 amino acids.

We excluded protein interactions with these ambiguous proteins. We also excluded those proteins that appear in pairwise interactions with both chaperones and non-chaperones. Although this step affected only 30 of the 175 chaperone-binding proteins in the bacteria group, for eukaryotes these numbers increased to 330 out of 574 and 505 out of 589 for metazoan proteins.

In addition, we compared the sequences of these unambiguously determined chaperone-binding and non-chaperone-binding proteins by Blastp and excluded those proteins in each group that matched a protein in the other group with at least 90% sequence identity. This step affected 0, 4, and 15 proteins in the bacteria, unicellular eukaryotic and metazoan protein group, respectively.

### Determining the percentage intrinsic disorder of interacting proteins

For all the interacting proteins in the three taxonomic groups we determined the percentage intrinsic disorder by counting all the disordered amino acids as predicted by IUPred [27],[28], dividing it with the total length of the protein and multiplying it with 100. We have selected IUPred for predictions because it has not been trained on potentially error-ridden data of disordered proteins. Rather, this algorithm estimates the total pair-wise interresidue interaction energy of sequences by applying low-resolution force-fields deduced from folded proteins. It has been observed that below a certain threshold the estimated energy is insufficient to overcome the large entropic penalty of folding, and (segment of) the protein cannot fold, but remains disordered. In this sense, IUPred score represents an assessment of the structural status of disordered proteins independent of prior rather heterogeneous data on IDPs.

### Distribution of the percentage disorder in the three taxonomic groups

For all the interacting proteins in the three taxonomic groups we determined the distribution of the percentage disorder of both the chaperone-binding and non-chaperone-binding proteins, by counting the number of proteins in each disorder range, with increments of 5% disorder. We actually used the percentage disorder values, by dividing the number of proteins for each range with the total number of proteins in that group and multiplying it with 100 (so that the area under each distribution curve adds up to 100).

### Identifying interacting and non-interacting proteins in *E. coli* and yeast

We also determined the percentage disorder distribution of proteins interacting with others and of those that do not seem to interact with any other protein in both *E. coli* and yeast. In this instance we did not focus on pairwise interactions but considered only SwissProt proteins (4931 *E. coli* and 6163 yeast proteins in SwissProt as of March, 2007) as only the latter have reliable annotations. In addition to any interaction data about a particular protein in IntAct we considered any protein in *E. coli* or yeast an interacting protein if it had the keywords ‘interaction(s)’ or ‘protein binding’ in its annotation.

### Propensity for chaperone-binding normalized to propensity for general partner binding

**Figure 1:**
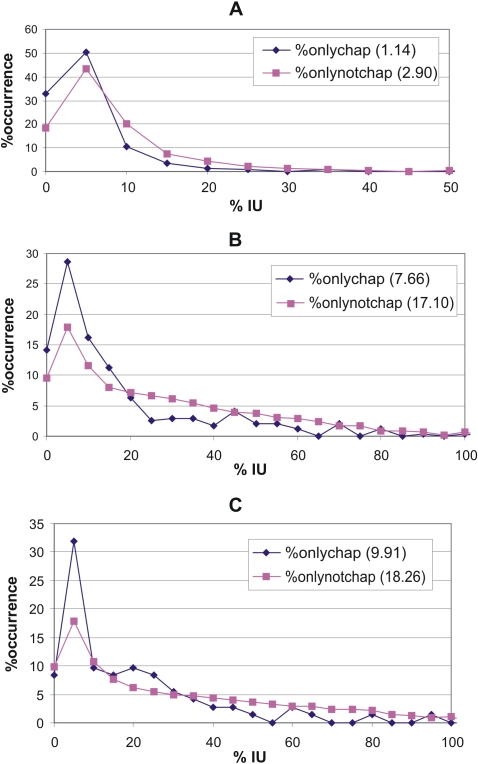
Distribution of the percentage of intrinsic disorder of chaperone-binding and non-chaperone-binding proteins in the three taxonomic groups. All the proteins detected in any kind of pairwise interactions in the IntAct database were taken into consideration. The percentage intrinsic disorder for each protein was calculated from disorder predicted by IUPred. The occurrence in each disorder range (with increments of 5% disorder) is given in % values, too, so that the area under each disorder curve amounts to 100. The median disorder values for the two sets of proteins are indicated in parentheses. (A) Bacteria (mostly *E. coli*). (B) Unicellular eukaryotes (mostly yeast). (C) Metazoa proteins.

**Figure 2:**
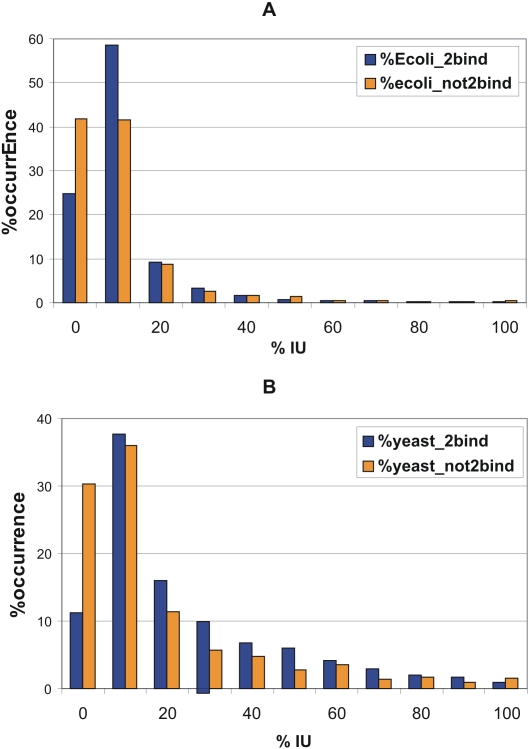
Distribution of the percentage intrinsic disorder of protein-binding and non-binding proteins in *E. coli* and yeast proteins in SwissProt. The occurrence in each disorder range is given in % values, too, as in Figure 1 (but with increments of 10% disorder). (A) All binding (2907) and non-binding (2015) *E. coli* proteins in Swissprot; (B) All binding (3630) and non-binding (2200) yeast proteins in Swissprot.

We divided all the proteins in *E. coli* and also in yeast into equal-size groups, bins, depending on their disorder. For each bin we calculated the ratio of chaperone-binding to non-chaperone binding proteins (Figure 1A) and divided it with the ratio of binding to non-binding proteins (Figure 2A). Normalization was carried out by the formula **R** = (**N**_chapb_/**N**_non-chapb_)/(**N**_bind_/**N**_nonbind_), where

1. **N**_chapb_ : number of chaperone-binding proteins in a bin;
2. **N**_non-chapb_: number of non-chaperone-binding proteins;
3. **N**_bind_: number of proteins binding at least one more protein;
4. **N**_nonbind_: number of proteins, not known to bind any other protein
5. **N**_chapb_+**N**_non-chapb_+**N**_nonbind_ = constant for each bin (*E. coli*: 446; Yeast: 572)

### Pfam-domain occurrence in proteins in pairwise interactions

We analyzed the interacting proteins in all the three taxonomic groups for Pfam domain occurrence [29]. We ran Blastp [26] with the proteins in pairwise interactions as queries against the database of Pfam-A domain sequences [29]. The e-value cutoff was set to 1e-5 and we took into consideration only the best match for each protein as we wanted to know only if the protein in question has a globular part or not.

## Results

### Disorder of chaperone-binding and non-chaperone-binding proteins in 3 taxonomic groups

In Figure 1 the percentage distribution of the intrinsic disorder (as predicted by IUPred, [27],[28]) of chaperone-binding, and non-chaperone binding proteins is presented. Figure 1A, 1B, and 1C present data regarding bacterial, unicellular eukaryote- and metazoa proteins, respectively, with the median values of disorder for each set also indicated. In bacteria and unicellular eukaryote the distributions of the two sets of proteins are significantly different according to chi-square tests, with p-values 0.01 and 1e-05, respectively, whereas in metazoa the difference between disorder distributions is not significant, even though the median value for non-chaperone-binding proteins is almost twice that of the chaperone-binding proteins (18.26% vs. 9.91% median disorder). The lack of significance is most certainly due to the small number (72 altogether) of the chaperone-binding proteins in this category. (If we doubled the numbers in this category, which would not change the distributions in Figure 1C, we would end up with a significant difference with a p-value <0.005). The overlaps between the chaperone-binding and non-chaperone-binding proteins in the different taxonomic categories are shown in Table 1. It is also clear from the table that the ratio of shared proteins (expressed in the percentages of all chaperone-binding proteins in Table 1 increases with the increasing complexity of the studied organisms. Median values of the disorder of chaperone-binding and non-chaperone proteins also underscore that the latter has a larger disorder in all three taxonomic groups. For all three taxonomic groups the median values of non-chaperone-binding proteins are about twice as much as for chaperone-binding proteins (Figure 1).

**Table 1:** The number of chaperones, chaperone-binding and non-chaperone-binding proteins, and the overlap between the last two in the 3 taxonomic groups

Group	Chaperones	Chap-binding	Non-chap binding	Binding both
Bacteria	66	175	719	32 (18%)
Unicellular Eukaryota	79	574	3863	330 (57%)
Metazoa	148	589	14674	505 (86%)

The percentage numbers in parentheses denote the ratio of shared/chaperone-binding proteins.

### Disorder is different for binding and non-binding proteins in *E. coli* and yeast

In the previous section we demonstrated that disorder shows anti-correlation with chaperone binding. We thought it is of interest to see if this reflects the general dependence of propensity for partner binding. To this end, we predicted the disorder distribution of all *E. coli* (Figure 2A) and yeast (Figure 2B) proteins known to be, or not to be, involved in pairwise interactions. For both organisms there is a clear-cut difference in disorder between binding and non-binding proteins most apparent at smaller values of disorder, with binding proteins being more disordered. For example, while there is practically no difference between proteins with zero disorder and with a maximum disorder of 10% for non-binding proteins in *E. coli* (both with about 40% relative occurrence), the values are sharply different for binding proteins: nearly 60% of all binding proteins have a disorder in the range of 0–10%, but only 25% of all binding proteins possess 0% disorder. The values are similar for yeast, with an even greater discrepancy between the two groups of proteins for this range of disorder. By a chi-square test, the two distributions of binding and non-binding proteins are significantly different, with p-values <1e-14 for both *E. coli* and yeast. This difference can be clearly attributed to the close link between intrinsic disorder and the involvement of proteins in physical interactions.

### The propensity of chaperone-binding normalized with propensity of binding

**Figure 3:**
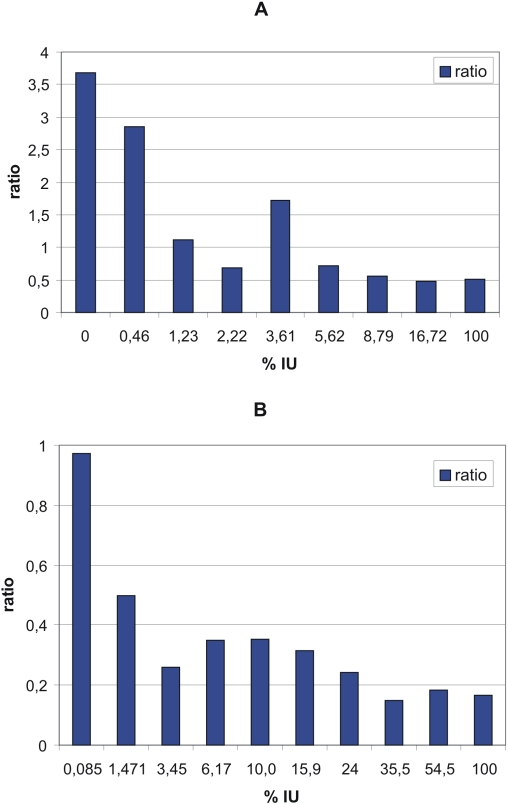
The propensity of chaperone-binding normalized to protein-binding for *E. coli* and yeast proteins as a function of disorder. The ratio of chaperone-binding and non-chaperone-binding proteins was divided by the ratio of protein-binding and non-binding proteins for each bin. Each bin contains the same number of proteins. The numbers on the X-axis indicate the upper values of the disorder range for each bin. (A) All *E. coli* proteins in SwissProt. (B) All yeast proteins in SwissProt.

Comparing Figures 1 and 2, one can conclude that both protein-binding and chaperone-binding vary as a function of intrinsic disorder, and next we asked how the tendency of chaperone-binding is related to the tendency of general partner-binding. Thus, in Figure 3A we proceeded in the following way: we divided all the proteins in *E. coli* into equal-size groups, bins, depending on their disorder. For each bin we calculated the **R** ratio of chaperone-binding to non-chaperone binding proteins (Figure 1A) and divided it with the ratio of binding to non-binding proteins (Figure 2A), as detailed in the *Methods* section.

In Figure 3B, we did the same for yeast proteins. The result in both cases is an almost monotonously decreasing function of protein disorder: i.e. normalized with binding propensity (which increases with increasing disorder) the propensity to bind a chaperone clearly decreases with increasing disorder.

### Occurrence of Pfam domains in chaperone-binding and non-chaperone binding proteins

The results obtained thus far indicate that disordered proteins tend to avoid chaperones, whereas ordered proteins prefer chaperones as binding partners. Percentage disorder within a protein, however, does not adequately distinguish between proteins with or without globular domains, which are potential chaperone binding sites of a protein. To clarify on this point, we decided to select and observe the chaperone binding of those proteins, which have at least one globular domain. As the Pfam domain collection contains mostly globular proteins (85% of them contains 10% disorder at the maximum (unpublished results)), the presence of a Pfam domain should represent the feature decisive for the need of chaperone-binding. To confirm this, we analyzed all the proteins in pair-wise interaction by Blastp against all Pfam domains. We found that for chaperone-binding proteins in unicellular eukaryotes the ratio of Pfam-lacking (i.e. those proteins where no Pfam-domain match was found) and Pfam-containing proteins was 0.380±0.10 (65 over 176 proteins) but for non-chaperone-binding proteins this ratio was 0.504±0.05 (1174 over 2356 proteins). In metazoa proteins the ratio of Pfam-lacking and Pfam-containing proteins for chaperone-binding proteins was 0.271±0.09 (16 over 59 proteins), whereas for non-chaperone-binding the same ratio was 0.45±0.01 (4387 over 9734 proteins). (By a chi-square test to compare the different proportions for both taxonomic groups, we found that these differences in ratios did not achieve statistical significances, but suggested clear tendencies. The lack of strict statistical significance is due mostly to the large differences in the number of chaperone-binding and non- chaperone binding proteins.) Thus, these observations were in line with our expectation that chaperone-binding proteins tend to have more often globular part(s), such as a Pfam domain, which require chaperone binding to help fold. Probably due to limitations in the number of known chaperone-binding proteins, we could not observe such a favorable difference between the ratios in bacteria (chaperone-binding proteins: 0.107 [14 over 131 proteins]; non-chaperone-binding proteins: 0,063 [40 over 636 proteins]) However, the disorder for all the 14 chaperone-binding bacterial proteins without a Pfam domain is below 31% therefore they can easily contain a globular domain. This is further supported by the observation that the name of 13 of the 14 Swissprot proteins in question starts with a ‘y’, indicating a largely uncharacterized bacterial protein in SwissProt.

### Ratio of exclusively chaperone-binding and non-exclusively chaperone-binding proteins

**Figure 4:**
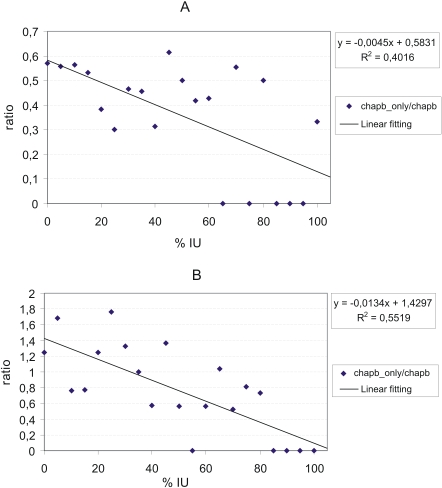
Intrinsic disorder of exclusively chaperone-binding and non-exclusively chaperone-binding yeast and metazoa proteins. The ratio of proteins which only bind chaperone to those which also bind non-chaperone partner(s). The numbers were counted for each disorder range (with increments of 5% disorder), then normalized to 100% for both the exclusively chaperone binding and non-exclusively chaperone binding proteins. In the last step the ratio of the two percentage numbers was calculated for each disorder range. A linear trendline is fitted to the data points—the equation of the trendline is indicated, with the resulting R^2^ value. (A) All yeast proteins. There are altogether 461 chaperone binding and 228 exclusively chaperone binding proteins in yeast. (B) All metazoa proteins. Altogether there are 497 chaperone binding proteins, of which 68 are exclusively chaperone binding.

Our explanation for all previous data is that ordered proteins/domains require chaperones as binding partners to assist their folding, whereas disordered proteins/regions either do not need chaperones or they need them for some other aspect of their function. From the limited information of relevance in the literature, we expect it might be rather for helping to integrate into larger complexes. The corollary of this suggestion is that IDPs that bind a chaperone are more likely also to bind another partner, than ordered proteins. As there is considerable overlap between the chaperone-binding and non-chaperone binding proteins, we thought to address this issue by determining if there is any difference in the disorder of those proteins that bind only chaperones and those that bind both chaperones and other types of proteins. The results are shown in Figure 4. For both yeast (Figure 4A) and metazoa (Figure 4B), there is a decreasing number of exclusively chaperone-binding proteins with increasing disorder. In the case of metazoa, none of the proteins with more than 80% disorder are exclusively chaperone-binding. There is a similar tendency for yeast proteins, too, with somewhat lesser R-value.

## Discussion

The major finding of our analysis is that predicted disorder of proteins negatively correlates with binding to chaperone partners, i.e. IDPs in the cell tend to avoid being bound to chaperones. This statement applies to a prokaryote, *E. coli*, a unicellular eukaryote, *S. cerevisiae*, and also to metazoa. The effect may be correlated with the presence of ordered domains, as observed with Pfam domains, although due to scarcity of data in two systems we could not draw a general conclusion. Further, binding of chaperones to disordered proteins is frequently accompanied by binding to other proteins, which suggests that IDPs use chaperones not for folding, but for assistance with association with other proteins. Even in cases where statistical significance is low, our data strongly discredit the original hypothesis that IDPs would be preferentially bound and protected by chaperones. These observations have numerous ramifications, as discussed next.

The first implication is that the very week correlation of protein disorder with intracellular degradation rate [13], is apparently not a general consequence of protection of IDPs by chaperones. Because IDPs *in vitro* are orders of magnitude more sensitive to proteolysis than globular proteins, this observation demands some other, general explanation, such as protection by protein-protein interaction or tight control of proteolytic systems. In fact, many functions of IDPs directly invoke their involvement in protein-protein interactions [2],[3],[30], and hub proteins with multiple interacting partners have an elevated level of disorder [31]–[33]. As a matter of fact, this may suggest that many interacting partners of proteins may also act in a compensatory or assisting fashion, given their potentially very high intracellular concentrations. Although this is not in the focus of the current work, our results might promote the idea of the extension and generalization of the chaperone concept. An additional point is that many intracellular proteases are known to be regulated and thus not to discriminately degrade their substrates. The mechanisms involve pro-enzyme activation (e.g. caspases), intracellular localization (e.g. lysosomal proteases) or ubiquitination (e.g. proteasome), among others. This might actually relieve chaperones from the duty of guarding IDPs, which might have been a key factor in the spread and functional success of IDPs.

Another pertinent issue is the structural ramifications of the noted preference of ordered proteins for chaperone partners. It is a commonplace that the 3D structure of a protein is determined by its amino acid sequence, but folding, in particular in the crowded intracellular environment of the cell, occasionally requires guidance by chaperones [17]. This, however, should be reflected in their need of chaperones during folding, not in the fully folded state, studied in the high-throughput interaction studies referred to. Thus, their preference for chaperones must reflect their tendency to transiently unfold and recruit a chaperone to assist refolding. In the case of IDPs, current *in vitro* observations suggest that they need no assistance for folding, i.e. they can reach the native-state ensemble from a highly denatured state spontaneously. The observation that IDPs are often heat resistant, and remain fully functional after treatment by boiling temperatures, bear witness to this point [4],[11],[34],[35]. This holds true also for IDPs that are not fully disordered, but have short-range [5],[36] and/or long-range [37]–[39] organization. As a first approximation, we may take this as an indication that a similar situation applies *in vivo*, i.e. IDPs spontaneously acquire their native ensemble of structures after synthesis. A key point here, however, is that chaperones might not only be needed for assisting proper folding, but also for preventing aggregation from a partially folded/misfolded state. It is thought the open and exposed character of IDPs makes them particularly vulnerable to aggregation, but their special amino acid composition itself counters the threat. In fact, IDPs are usually highly charged, they contain a high percentage of the structure-breaking Pro residue, and are low in hydrophobic residues, which all act against aggregation and subsequent amyloid formation [4]. Further, they have special sequence features built in to prevent aggregation, as noted in the case of the polyGln region of huntingtin [40]. Since IDPs do show some tendency to interact with chaperones, it seems appropriate to suggest that one prime reason for these interactions is to prevent amyloid formation. This has been explicitly stated in the case of the yeast prion Ure2 interacting with Hsp40 [41], α-synuclein interacting with Hsp70 [20],[42] and expanded polyQ regions interacting with both Hsp40 and Hsp70 [43]. Interestingly, in one case it has been suggested that the chaperone in fact does not interact with the IDP, but rather a prefibrillar intermediate, which may be a general phenomenon among other IDPs as well [42].

The final point that deserves closer inspection is the possible functional implications of chaperone binding of IDPs, given their lack of need of assistance for folding to a functional state. Two conceivable requirements are transport through physiological membranes and assistance for partner binding, i.e. assembly of complexes. In the case of transport through membranes, globular proteins partially unfold to a molten-globule state competent with translocation through the membrane and refold at the other side by the help of other chaperones. IDPs in principle do not need such help as they are already in a translocation-competent structural state [44]. As to their binding to other partners, and the subsequent assembly of complexes, IDPs in fact often carry out their functions by protein-protein interactions [4],[5], also shown by that the average disorder increases with increasing size of complexes [45]. However, IDPs have been observed *in vitro* to be very effective in binding, primarily manifested in binding to the partner at an increased speed [4],[46]. Their avoidance of chaperones, in general, may be related to this. When they do bind chaperones, however, the reason might be that *in vivo* assembly of large complexes may be slowed by non-specific interactions, in the case of which chaperone assistance may be of help.

In conclusion, we report here that IDPs in general require less assistance from chaperones than ordered, globular proteins. The explanation of this negative preference probably stems from the fact that IDPs are rather autonomous in folding, and require little assistance in function. Their liability for amyloid-type aggregation, and involvement in the assembly of large complexes, however, do explain their occasional binding to chaperones. Further studies may address at the level of individual proteins if this is in fact the case.
